# The Contributions of Nina Fedorovna Talyzina to Research Developed in Brazilian Postgraduate Programs

**DOI:** 10.11621/pir.2023.0308

**Published:** 2023-09-30

**Authors:** Luiz Fernando Pereira, Isauro Beltrán Núñez, Alison Luan Ferreira da Silva, Maria Helena de Andrade, Frank Madson Araújo de Melo, Marcus Vinicius de Faria Oliveira

**Affiliations:** a Federal University of Rio Grande do Norte, Natal, Brazil; b Federal Institute of Education, Science and Technology of Rio Grande do Norte, Natal, Brazil

**Keywords:** activity theory of learning (ATL), Brazilian research, bibliographic review, cultural-historical school (CHS), developmental didactics

## Abstract

**Background:**

Nina Fedorovna Talyzina was a Russian psychologist, whose theories have been applied in educational research in many countries around the world, including Brazil. Her name is mainly connected to the Activity Theory of Learning (ATL), which has been dubbed the Galperin-Talyzina system of developmental didactics.

**Objective:**

Investigate how N.F. Talyzina’s ideas are applied in dissertations and theses developed in postgraduate programs in Brazil.

**Design:**

Our research was a bibliographic review which used the state of the question method to examine how Talyzina’s ideas are applied in Brazilian academic publications. Data were gathered from three responsible databases – the Brazilian Digital Library, the CAPES Catalogue, and the Institutional Repositories (1987–2022). The method of content analysis was used for data analysis, according to pre-determined categories.

**Results:**

We found a prevalence of research based on the methodology of formative experiments carried out at different educational levels. Skills, scientific concept- formation, and problem-solving were the objects of investigation in most of the studies. The ideas proposed by L.S. Vygotsky, A.N. Leontiev, P.Ya. Galperin, and V.V. Davidov were expressed through references to Talyzina’s work as the theoretical basis of many studies, thus evidencing a crucial dialogue with the Cultural-Historical School (CHS).

**Conclusion:**

Our study points out the growing interest in Talyzina’s ideas, specifically her Activity Theory of Learning, which can be attributed to comprehensive dialogues with the ideas of L.S. Vygotsky and A.N. Leontiev which predominate in Brazilian research.

## Introduction

Nina Fedorovna Talyzina (1923–2018) was a researcher and professor at the Department of Educational and Pedagogical Psychology at the Faculty of Psychology at M. Lomonosov Moscow State University. She has been recognized as one of the most noted Soviet/Russian psychologists, especially in the area of educational psychology. She studied learning processes and critically analyzed both behaviorist and cognitive approaches, concluding that both imposed limitations on students’ psychological development (Talyzina, 2023a). Her work is known in Brazil as the Activity Theory of Learning (ATL), and aims to understand the nature and process of assimilation, and creative appropriation of subjects in the school context (Núñez, Fariñas & Ramalho, 2020). However, as Solovieva & Quintanar (2015) state, her scientific ideas are more extensive since they cover a significant diversity of topics that were part of her scientific career at the aforementioned world-renowned university.

The ATL (Talyzina, 2023a) has been widely used to support research in education and the teaching-learning processes in the school context in several countries. In the opinion of Semenov (2014a), it is the methodological basis for psychological and pedagogical research, as well as for practical pedagogy. It is a theory with a solid psychopedagogical component, supported by a significant volume of research results in classroom contexts, and has become a reference for studying teaching and learning processes (Solovieva & Quintanar, 2020). The importance of this theory is also emphasized by authors such as Gabay (2014), and Semenov (2014a). In the opinion of Zhdan (2013), the ATL is possibly the richest of all modern psychological theories, as it is a fundamental theory of human activity as an explanatory principle.

According to N.F. Talyzina, the foundations of the ATL were laid in the theoretical system of P.Ya. Galperin (Talyzina, 2002). It is true that her theory is not a specific application or extension of Galperin’s ideas (which comprised a more general psychology), but rather a new theory with its own identity, although it strongly shares Galperin’s theoretical and methodological assumptions, as Gabay explained (2012). The ATL also has its foundations in A.N. Leontiev’s Activity Theory, as N.F. Talyzina herself recognized. In developing her theory, she demonstrated the possibility of connecting the contributions of P.Ya. Galperin and A.N. Leontiev in order to understand teaching processes (Talyzina, 2002), and highlighted their contributions to improving psychodiagnosis (Talyzina & Karpov, 1987; Talyzina, 2020), psychological mechanisms of generalization (Talyzina, 2001), motivation (Talyzina, 2017), qualitative parameters of the orienting base of action (Talyzina, 2002), and the question of the psyche as an activity (Talyzina, 2023a). Her theory also brought important contributions to the activity approach in Psychology (Talyzina, 1984).

N.F. Talyzina had (and still has) an important influence on the training of researchers in several countries in Europe, Asia, Africa, and Latin America. These include countries such as Spain, Finland, Japan, Germany, Mexico, and Cuba, where she developed in-person activities for several years. In these and other countries, many of her works have been translated and published. In the case of Brazil, her influence on educational research began in the 2000s (Núñez et al, 2020).

The appearance of N.F. Talyzina’s research in postgraduate programs in Brazil, according to Puentes & Longarezi (2020) and Longarezi (2020), was associated with the growth of studies based on Activity Theory. These studies can be understood from the perspectives of two major research groups: a) the Cultural-Historical Activity Theory (CHAT) perspective based on the works of L.S. Vygotsky, A.N. Leontiev, J.V. Wertsch, and Y. Engeström; and b) the perspective of the Activity Approach based on the works of L.S. Vygotsky, A.N. Leontiev, P.Ya. Galperin, V.V. Davidov, N.F. Talyzina, L.V. Zankov, V.V. Repkin, and D.B. Elkonin on the paradigm of developmental teaching. This second perspective is held by a significant group of researchers in Brazil, a country in which the Galperin-Talyzina framework is recognized as a system within the Cultural-Historical School (CHS) (Puentes & Longarezi, 2020).

Several theoretical studies and research reports based on the contributions of N.F. Talyzina have been published in Brazil (Feitosa, Mendoza & Delgado, 2022; Farias & Rego, 2020; Marco, Cecho & Lopes, 2021; Moraes & Borges, 2021). It is important to highlight the point of view of the researchers who integrate the ideas of N.F. Talyzina, P.Ya. Galperin, and especially those of L.S. Vygotsky and A.N. Leontiev. In this sense, we also emphasize the publication of the book *Developmental Teaching: The Galperin-Talyzina Theoretical System,* published by Editora Científica Digital in the year 2021, which consists of 10 chapters written by authors from various countries, such as Brazil, Mexico, Cuba, and Russia. Also noteworthy is the scientific journal *Obutchénie*, which is published by the Federal University of Uberlândia (UFU); this journal, founded in 2017, appears quarterly and has frequently published papers linked to N.F. Talyzina’s ideas.

The increasing interest in Talyzina’s ideas constitutes the main justification for conducting this study of the state of the question involving academic publications. As Ortiz Torres (2018) argues, this type of study is important whenever research on an issue is growing and the volume of information is increasing. Thus investigations may have reached the density where systematization of what has been produced is needed, as well as the identification of emerging aspects necessary for continuing its growth and developing new productive lines of research.

## Methods

This study consisted of an investigation of the state of question type (Ferreira, 2002; Nóbrega-Therrien & Therrien, 2004), considered from the perspective of a bibliographic review, to analyze the inclusion of Talyzina’s ideas in dissertations and theses produced in postgraduate programs in Brazil.

Romanowski & Ens (2006) and Ortiz Torres (2018) have highlighted regular procedures for bibliographic research through applying the method of the state of the question. The method includes: 1) choice of descriptors; 2) definition of databases to be consulted; 3) definition of criteria for data selection; 4) collection of data to constitute a corpus; 5) analysis of content; and 6) data organization and analysis by categories.

### 1. Choice of descriptors

We adopted the following expressions as descriptors or keywords: Talyzina; Activity Theory; Activity Theory of Learning; Theory of Approximation of Activity; and Talyzina’s Theory of Teaching Direction. These expressions seemed representative of those needed to find academic publications related to the research objective.

### 2. Definition of the databases

We used three databases for the search, as described below:

- the Brazilian Digital Library of Theses and Dissertations (BDTD). This database has been constituted and maintained by a large collaborative effort involving 127 Brazilian research institutions, which freely publicize thousands of dissertations and theses, allowing wide dissemination of research produced in Brazil. The BDTD has received its data from postgraduate programs since 1987. (Link: https://bdtd.ibict.br/vufind/)
- the Thesis and Dissertations Catalogue of The Coordination of Improvement of Higher Education Personnel (CAPES). CAPES is a governmental agency for regulating, funding, and evaluating all postgraduate programs in Brazil. It was launched in 2002 and brings together all the dissertations and theses defended in Brazil or by Brazilians abroad. Link: http://catalogodeteses.capes.gov.br/catalogo-teses/#!/
- Repositories of Higher Education Institutions. Many dissertations and theses produced in new research institutions that are not yet part of the BDTD or CAPES Catalogue are stored in their institutional databases. For data collection, there are unlisted Higher Education Institutions because their institutional repositories were not found or not available in the period of investigation.

In this work, we intended to use the BDTD and CAPES databases in a complementary way, supplemented by data found in some institutional repositories, which allow us to obtain a complete sampling of the Brazilian academic publications (dissertations and theses).

### 3. Definition of the criteria for data selection

Initially, we selected from the dissertations and theses available in the databases — including the first and most recent studies developed in Brazil (2003 to 2022) — those using Talyzina’s theory as a foundation in their theoretical matrix, as well as those in which this theory was used in a complementary way. 

### 4. Collection of data to constitute the corpus

Using the presented descriptors and criteria (items 1 and 3), we found a total of 61 dissertations (master’s level) and 33 Ph.D. theses, totaling 94 academic publications using Talyzina’s ideas at the graduate level. This overview reveals a part of what has been produced in Brazilian research on this author’s ideas.

### 5. Analysis of content

Data analysis was performed using the content analysis method (Bardin, 2016; Franco, 2018; Amado, 2000). According to Bardin (2016), content analysis comprises two basic procedures:

- *Exploration of the material.* Procedures were carried out by reading titles and abstracts of the 94 literary productions to get an overview of their general characteristics.
- *Data processing and interpretation.* This refers to generating inferences on the corpus, getting results that allow us to identify the objectives of the investigation. The following categories for interpretation of the data were constituted in light of the responses for such characteristics: 1) exhaustive, which means that all elements of the replies can be included in them; 2) mutually exclusive, which means that each element can only be part of a single category; 3) objective, meaning that they must be defined in a precise manner that avoids ambiguity; and 4) relevant, meaning that they must be appropriate to the research objectives (Ortiz Torres, 2018).

For the analysis, the data were grouped and processed in three steps:

*Step 1.* Initially, the dissertations and theses were categorized into two groups depending on whether Talyzina’s ideas were applied as the main or supplementary reference of the theoretical matrix. Subsequently, the academic publications were divided into those in which Talyzina was the main author cited (06) and those in which Talyzina was a supplementary author discussed (88).

*Step 2.* All 94 productions were sorted according to the following categories: 1) type of academic production (master’s or Ph.D.); 2) year of production; 3) educational level of the participants (Primary, Elementary, Middle or High School, Higher Education); 4) subjects related to the study; 5) type of study; and 6) study object and references from authors related to the CHS.

*Step 3.* A characterization of the specific references that supported the academic publications: Talyzina’s works used in the study (book and/or paper) and references from other authors who are part of the CHS.

### 6. Data organization and analysis by categories

The Microsoft Excel software was used to organize the information from each academic publication. The data set was analyzed by the research authors who elaborated the tables which made it possible to perform quantitative and qualitative analyses based on descriptive statistics; *i.e.,* the distribution of frequencies or percentages from data presented in tables, according to the research objective (Hair et al., 2014). Analyses were individually performed by different researchers, who shared and discussed their results to reach a consensus at the conclusion of the research, which increased the reliability of the findings (Flick, 2020).

## Results

The results were presented in relation to the study questions formulated from the general objective, with the purpose of showing the relevant aspects of the data.

### The academic production of studies based on N.F. Talyzina’s theoretical ideas

The search for academic publications (master’s and Ph.D. levels) linked to the theoretical ideas of N.F. Talyzina in the consulted databases, allowed us to identify a total of 94 academic papers, 61 of which were at the master’s level and 33 at the Ph.D. level. For a better understanding of the place of N.F. Talyzina’s ideas in the theoretical references that support the studies, the publications were classified into two groups: a) those in which the author’s ideas formed the core of the theoretical matrix; and b) those in which the ideas were constituents of the references, without representing the core. The results of this classification are shown in *Table 1.*

**Table 1:** Academic publications per postgraduate level

Publication	Talyzina’s ideas used in the central core of the theoretical matrix	Talyzina’s ideas related to the core of the theoretical matrix	Total
**master’s level**	4	57	61
**Ph.D. level**	2	31	33
**Total**	6	88	94

According to *Table 1*, the highest percentage corresponded to master’s dissertations (64.9%) compared to Ph.D theses (35.1%). In turn, the number of academic publications centered on the ideas of N.F. Talyzina’s ATL was still smaller; that is, there was a small number of publications in which this theory was the central subject of the research. Consequently, the highest percentage (93.6%) were publications in which N.F. Talyzina’s ideas, although not constituting the core, were relevant in the construction of the theoretical matrices.

### The academic publications linked to N.F. Talyzina in Brazil year by year

**Figure 1.**
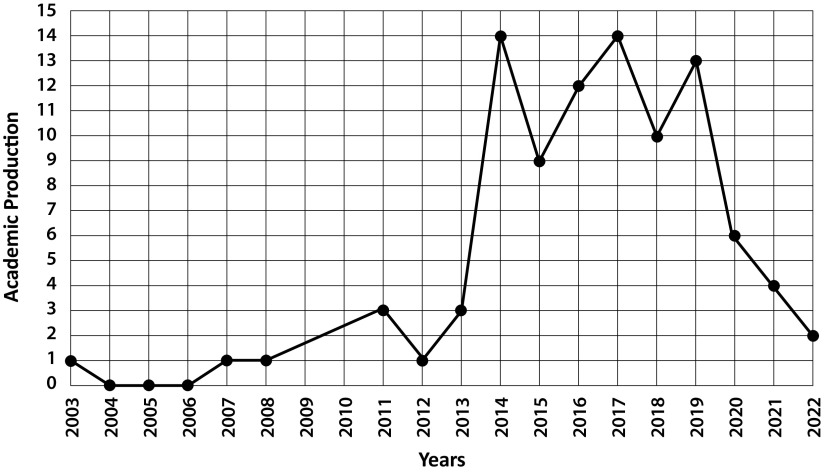
The academic publications (master’s and Ph.D. levels) produced per year

In *Figure 1*, we can observe a growth trend in academic publications based on N.F. Talyzina’s ideas in Brazil over the period 2003–2019.

The decrease from 2019 may be linked to the non-inclusion of new academic publications in the databases consulted for that period.

### The Activity Theory of Learning in the Theoretical Foundations of Academic Publications

The study also made it possible to identify the presence of the ATL and its relationship with other theories of the CHS in academic publications. *Table 2* shows the results of this identification, linking the theories cited to their main authors.

**Table 2:** Main authors of academic publications

Author	Ph.D. level	Master’s level	Total
Galperin-Talyzina	11	48	59
Vygotsky-Talyzina	17	9	26
Talyzina	2	4	6
Leontiev-Talyzina	3		3
**Total**	33	61	94

We can observe in *Table 2* that of the six academic publications focused on N.F. Talyzina’s ATL, the greater quantity (83.3%) appear to be associated with P. Ya. Galperin’s Theory. In turn, considering the total publications, the number of dissertations and theses (62.7%) that focused on P.Ya. Galperin’s theory and elaborated it with N.F. Talyzina’s Theory was significant; that is, there was a predominance of what has been identified in Brazil as the Galperin-Talyzina system. In other academic publications, N.F. Talyzina’s Theory appeared as a complement to the theoretical configurations associated with Vygotsky’s Cultural-Historical Theory and A.N. Leontiev’s Activity Theory.

### Educational levels and subjects addressed in academic publications

The educational levels and disciplines in which research for academic publications were carried out reveal certain foci of attention in postgraduate studies that are based on the ATL of N.F. Talyzina.

As can be seen in *Table 3*, studies were carried out at all levels of education, with the main emphasis on higher education (34.0%) and high school (26.5%), and a lesser emphasis on middle (16.0%) and elementary school (13.8%). In the case of higher education, the research focused on teacher training. Regarding the subjects of the school curriculum, the area of mathematics education (35.1%) was the most popular, followed by the teaching of physics (13.8%), chemistry (12.8%), science in elementary education (9.6%), and biology (4.2%). The other areas of the school curriculum comprised 20.2% of the publications, and were distributed in small proportions among disciplines such as history, physical education, and others.

**Table 3:** Academic publications involving different subjects and educational levels

Subject	PE	ES	MS	HS	HE	NA	Total
Mathematics		6	10	5	10	2	33
Physics				10	3		13
Chemistry				3	7	2	12
Sciences	1	3	3	1	1		9
Portuguese		3	1	1			5
Biology				2	2		4
Multidisciplinary					3	1	4
Not applicable	1	1			2		4
History			1	1		1	3
Physical Education					1	1	2
Computing				1			1
English					1		1
Marketing					1		1
Pedagogy				1			1
Educational Psychology					1		1
**Total**	2	13	15	25	32	7	94

*Note. PE: Primary Education; ES: Elementary School; MS: Middle School; HS: High School; HE: Higher Education; NA: Not Applicable.*

## The objects of study of the academic publications

The objects of study addressed in the academic research are presented in *Table 4.*

**Table 4:** Objects of study addressed in the academic publications

Objects of study	Frequency
Skills Formation	52
Formation of Concepts	36
Problem-Solving	27
Special Education (Deaf, Hearing Impairment, Down’s Syndrome, Deaf-blind, Hearing-visual Impairment, Blindness, Visual Impairment)	7
Reading and Writing	5
New ICT / Virtual Environments for Education	4
Experimental Activity	4
Creativity	3
State of Art	2
Assessment	1
Planning	1
**Total**	**142**

There was a clear preference for three objects of study: a) skills formation (36.6%); b) formation of concepts (25.3%); and c) problem-solving (19.0%). It is important to highlight that, although they occur in a smaller proportion, other very important topics for education appear, such as: special education (4.9%), difficulties in reading and writing (3.5%), and the use of new technologies (2.8%). Finally, creativity is an object that, despite its importance, is rarely addressed in these studies.

### Types of references cited in academic publications

Identifying and characterizing the most frequent references in the analyzed academic publications can provide important information about which of N.F. Talyzina’s ideas predominate as the basis for dissertations and dealing with the ATL. In turn, they can highlight the absence of some ideas which are important for understanding N.F. Talyzina’s thought. *Table 5* shows the references to N.F. Talyzina in the publications consulted by the authors (classified into books, book chapters, and papers).

**Table 5:** Type of references to N.F. Talyzina cited in the analyzed publications

Type of Reference	Citation Frequency	%
Book	226	91.5
Paper	16	6.5
Book Chapter	05	2.0
**Total**	**247**	**100**

As the table shows, the highest frequency of references was to Talyzina’s books (91.5%). In comparison, the author’s papers and book chapters were rarely cited.

*Table 6* shows the most cited books and the frequencies with which they were referenced in theoretical and methodological discussions and in discussions of the results of the ATL.

**Table 6:** The books written by N.F. Talyzina most consulted in the academic publications

Books	Frequency	Language
Psychology of learning	70	Spanish
Pedagogical psychology	45	Spanish
Activity theory applied to teaching	31	Spanish
The formation of mathematical thinking skills	25	Spanish
Lectures on the fundamentals of teaching in higher education	24	Spanish
Formation of students’ cognitive activity	13	Spanish

The books most used as references were: *Psychology of Learning* by Editorial Progreso, published in 1988, with a frequency of 33.6%, and *Pedagogical Psychology*, published in 2000, in Mexico, with a frequency of 21.6%, both in the Spanish language.

Although they have a low percentage of consultations, in *Table 7* we identify the main papers of Talyzina that circulate in postgraduate programs in education in Brazil.

**Table 7:** The most consulted papers written by N.F. Talyzina in the academic publications

Paper	Frequency	Language
Talyzina, N.F., Solovieva, Y., & Quintanar, L. (2010). The approximation of the activity in psychology and its relationship with the cultural-historical approach of	06	Spanish
L.S. Vygotsky. *Revista Novedades Educativas,* 230, 4–8. Talyzina, N.F. (2008). Psychological mechanisms of generalization. *Acta Neurolology*, 24(2), 76–88.	04	Spanish
Talyzina, N.F. (1968). Analysis of Galperin’s theory. *Revista Psicologia e Educación*, 5(10), 33–41.	02	Spanish

These are articles that complement the theoretical discussions presented in her books, which, in a way, explain the aspects being researched, such as the question of generalization and its psychological mechanism and foundations based on the contributions of the ideas of A.N. Leontiev and P.Ya. Galperin. The only book chapter written by N.F. Talyzina cited in the analyzed academic publications was: “The principles of Soviet psychology and problems of psychodiagnostics of cognitive activity.”

Núñez et al. (2020) consider it necessary to understand the ATL of N.F. Talyzina in the context of the contributions of relevant authors of the CHS. In *Table 8* these authors are presented in this aspect, differentiating between their use in the theoretical matrix, and in the subsidiary analyzes and discussions of the research results.

**Table 8:** References to authors related to the CHS in the academic publications

Author	Used only in the con#guration of the theoretical matrix	Used in the theoretical matrix and in the discussion of results	Total
P. Ya. Galperin	47	38	85
L. S. Vygotsky	48	29	77
A. N. Leontiev	58	10	68
V. V. Davidov	35	04	39
M. I. Majmutov	20	9	29

According to *Table 8*, in general, the references to P.Ya. Galperin’s theory are the most frequent (28.5%), followed by references to L.S. Vygotsky’s Cultural-Historical Theory (25.9%) and A.N. Leontiev’s Activity Theory (22.8%). The Theory of Developmental Teaching by V.V. Davidov (13.1%) and Problem Teaching by M.I. Majmutov (9.7%) appear in smaller proportions.

### Types of studies based on the Activity Theory of Learning

Another aspect analyzed in the state of the question method concerns what types of study were conducted which included Talyzina’s theory in the research configuration. They are shown in *Table 9.*

**Table 9:** Types of study in academic publications

Theoretical system	Empirical Study	Formative Experiment	Other	Total
Galperin-Talyzina	20	38	1	59
Talyzina	1	5	0	6
**Total**	21	43	1	65

*Table 9* presents the results of the types of research related to the Galperin-Talyzina and Talyzina theories. As can be seen, there is a predominance of formative experiments (according to the authors of the academic publications) in relation to other types of empirical studies.

## Discussion

The general objective of this study was to identify and characterize the influence of N.F. Talyzina’s ideas and, in particular, her ATL (Talyzina, 2023a) in academic productions at the master’s and Ph.D. levels in postgraduate education in Brazil. Its importance is due to the relevance of that theory to discussions of the activity approach in Psychology, as noted by Gabay (2014), Núñez et al (2020), Semenov (2014b), and Solovieva & Quintanar (2015).

As was previously observed, from the year 2006 onwards, there was a growth in academic publications in the area of education related to the ATL. This escalated in the period from 2012 to 2019, showing an interest in the theory as it has shown its potential for shaping education that contributes to the intellectual development of students in the school context. We link the decrease observed in the period from 2019 to 2022, as explained in an article by Núñez, Pereira, Amaral, Silva and Barros (2023), to the effects of the COVID-19 pandemic, which brought about the extension of the deadline for the completion of research in postgraduate programs in Brazil.

The total number of academic publications (94), when classified into those in which Talyzina’s theory was the structural foundation of the research (6), and those in which the theory was associated secondarily with other theories of the CHS (88), can lead us to state that the number of publications of the first type in Brazilian postgraduate studies is still small. Nevertheless, the existence of theoretical configurations in research that are associated with and in dialogue with this framework (one that expands the understanding of the teaching and learning processes along the lines of the CHS) is positive, since, as Zhdan (2018) states, it is a theory with a positive potential for the practice of education.

Our results showed that N.F. Talyzina’s ATL, as a reference in academic publications, appeared primarily in association with other theories, such as the P.Ya. Galperin’s Theory of the Planned Formation of the Mental Actions and the Concepts, and A.N. Leontiev’s Activity Theory. The ideas of N.F. Talyzina appeared in Brazil as a result of the growth of interest in authors from the CHS, in particular, L.S. Vygotsky and A.N. Leontiev, according to Longarezi (2020). In turn, there is a certain understanding of dialectical relationships in Brazil, in terms of contributions to looking at the ATL within certain didactic systems such as the Galperin-Talyzina system. The status of a didactic system which articulates the ideas of P.Ya. Galperin and N.F. Talyzina, is conceptually linked to what Davidov (1997) defines as a “pedagogical system” or “scientific-practical pedagogical.” This didactic system is discussed in Brazil as the Developmental Didactic System (Núñez et al, 2020).

Our research shows that the ATL was directed at all levels of Brazilian education, with an emphasis on high school and higher education. We identified the most prioritized disciplines as mathematics and the area of the natural sciences. Although the ATL references consulted in the research were mainly oriented to teaching children, the fact that the studies addressed the learning process of adolescents and adults reveals the breadth of the theory developed by Talyzina herself and her collaborators (Talyzina, 2023b). In a way, it reinforces a research trend in the area of mathematics, where N.F. Talyzina’s studies originated, as well as the development expressed in several publications. Prioritizing the area of natural sciences is linked, in a way, to the areas of research in postgraduate programs in which the academic publications were produced, which, in general, are in the areas of mathematics and science teaching.

A variety of objects of study was approached in the academic publications, especially the formation of skills and theoretical concepts and problem-solving applied to the teaching-learning process, preferably of mathematics and natural sciences. This scenario is similarly related to the experiences and training of professors who lead research groups in postgraduate programs with the largest amounts of scientific output.

The formation of concepts and skills, in addition to problem-solving, are relevant topics in N.F. Talyzina’s research. When considering learning as a special type of activity in the school context, knowledge is not separated from the action through which it is acquired and applied. As Talyzina (2023a) understood, action is the psychological mechanism for forming concepts.

It is important to point out that the objects of study related to special education are less frequently presented as an area of application of the theory: for example, research with students with Downs syndrome, and visual or hearing impairment. The ATL in Brazil, together with the contributions of L.S. Vygotsky, A.N. Leontiev, and recently P.Ya. Galperin, has opened up new possibilities for debates on special education, which has been historically guided by so-called constructivist references (Núñez et al., 2023).

Among the references to N.F. Talyzina which we consulted and used as theoretical foundations, we found six books to be among the most cited; they are translations into Spanish of classic texts by Talyzina, which express the essential aspects of organizing the teaching process under the ATL. Papers consulted in academic publications appeared in smaller numbers compared to books. Two of them discuss key issues of the ATL in relation to its theoretical foundations and relationship with the ideas of A.N. Leontiev and P.Ya. Galperin. Nevertheless, the limited number of publications by N.F. Talyzina in Brazil is evident. Translations into Spanish prevail, with the absence of Portuguese, which may influence the limited references to the ATL.

As we have shown, N.F. Talyzina’s bibliographical references were associated with those of other authors from the CHS. Among these authors, P.Ya. Galperin stands out, with a frequency of 85 times. In second place appears L.S. Vygotsky, who was referenced 77 times. References to A.N. Leontiev appeared 68 times. The works of V.V. Davidov and M.I. Majmutov appeared to a lesser extent. It is essential to highlight that problem-solving, according to Talyzina (2023a), is an important way to develop students’ motivation. Therefore, the publications based on the ATL proceed from this assumption in the organization of the teaching activity.

The works of L.S. Vygotsky have significantly influenced the training of researchers in the area of education, which may explain the number of times they were consulted. The next most influential is A.N. Leontiev (Leontiev, 2010). These connections between the mentioned theorists are necessary. In this direction, Solovieva & Quintanar (2021) emphasize that knowing Talyzina’s contributions to Psychology requires studying and learning the ideas of L.S. Vygotsky and A.N. Leontiev (we add those of P.Ya. Galperin) without which Talyzina’s texts would not be clear.

Finally, we presented important issues revealed in the analysis of the academic publications that based themselves on Talyzina’s theory.

An important amount of research has been based on experimental studies, mostly of the formative experiment type, on the Galperin-Talyzina system. In these studies, a focus on the management of learning processes according to the stages of Galperin’s Theory has been observed, without taking into account, in general, the qualitative parameters of the actions. Formative strategies articulate the learner’s external and internal actions, in terms of subject’s orientation, or orienting base. However, task systems oriented to the formation of mental actions are not created, in terms of the dimensions of orientation that support the execution and regulation of processes. The use of orienting bases, in general, of a particular type, was identified, which leads to some mistakes in the materialization of the orientation, essential in the processes of formation of mental actions. This situation shows that the studies are not considering P.Ya. Galperin’s theory as a system of psychological conditions that can guarantee the study of the formation of mental actions and concepts (Galperin, 2023), as elaborated by N.F. Talyzina’s ATL, as a reference for organizing and developing formative experiments.

In general, the following aspects were found, which revealed the contributions of N.F. Talyzina through the Galperin-Talyzina System to Brazilian postgraduate studies:

1. The types of student orientation towards action proposed by Talyzina are being used (Talyzina, 2023a); Talyzina expanded the ideas of orientation as the main function of the human psyche in Galperin’s Theory, by showing the possibilities of not just three types, as Galperin believed, but of eight, which resulted in an understanding of student orientation in the Zone of Proximal Development (Ilyasov, 2014). In the Brazilian works, it is not possible to identify which type of orienting base was used, according to the criteria established by N.F. Talyzina and P.Ya. Galperin. There is an intention to use orientation with students in relation to the execution and regulation of the processes of forming concepts, mental actions, and specifically problem solving.
2. The discussion of motivation for learning, based on external and internal motivations, their dynamics, and how they influence the process (Talyzina, 2017, 2023b);
3. Learning control processes (Talyzina, 2019), although the qualitative parameters of actions, and in the broadest sense, the management of teaching processes, are not explored.
4. The academic publications do not reflect other contributions by Talyzina, among them the mechanisms of generalization of activity (Talyzina, 2008). Although Talyzina’s theory is considered part of the Galperin-Talyzina Developmental Didactic System (Núñez et al, 2023), research has not been able to establish explicit relationships between teaching and students’ psychological development under the influence of formative experiences.
5. No studies were found on structuring curriculum proposals in professional training or basic education according to Talyzina’s ideas.

N.F. Talyzina criticized the curricula for training higher-level specialists for their one-sidedness, and developed new principles for training professionals with a broad profile. These studies can be interpreted in light of modern attempts to develop curricula based on general competencies of professional activity. In this sense, N.F. Talyzina’s contributions in the area of university education were diverse and relevant (Núñez, Fariñas & Ramalho, 2020).

## Conclusions

The research that took N.F. Talyzina’s ATL as a reference, in a way, showed that researchers are seeking to adapt and expand the ideas of L.S. Vygotsky, A.N. Leontiev, and P.Ya. Galperin in different contexts in Brazil. In these studies, the application of an important principle of N.F. Talyzina’s ideas is verified: the inseparable union between knowledge and action.

The results of the study showed that the ideas of N.F. Talyzina provide a foundation for dealing with problems in organizing the management of educational processes, modeling, and the formation of various types of activities, including the development of teaching objectives, organization of learning activities, development of concepts and skills, processes of control and regulation of learning by students, among others.

Talyzina’s theory of learning is strongly related to P.Ya. Galperin’s theoretical ideas, which in Brazil are considered the Galperin-Talyzina System. These studies are carried out at all levels of education, with emphasis on high school and higher education, focusing on the areas of science and mathematics teaching.

In general, academic studies in Brazil, although still specific, corroborate the advantages of the ATL in educational processes in the school context, showing its compatibility with the demands of education for all in the 21st century.

To carry out future studies, we suggest a more qualitative characterization of the research results mentioned in this paper, based on a meta-analysis, to better understand the contributions of academic publications in postgraduate studies in Brazil, in particular the singularities of the application of the Activity Theory of Learning in the context of education.

## Limitations

The categories of analysis reflect a certain subjectivity contributed by the participation of several researchers in this process. This limitation was partly reduced by training these researchers to discuss the meanings they attributed to the data, which contributed to a greater reliability of the analysis.
